# Impact of Parental Knowledge on Prevention Risk of Caries in Seville Children Between 6 and 14 Years Old, Applying the CAMBRA Protocol

**DOI:** 10.3390/children12070824

**Published:** 2025-06-23

**Authors:** Esther Pérez de Mora, José María Barrera-Mora, Marcela Arenas-González, Asunción Mendoza-Mendoza, David Ribas-Pérez

**Affiliations:** 1 Paediatric Dentistry, Faculty of Dentistry, Complutense University of Madrid, 28040 Madrid, Spain; estperde@us.es; 2 Orthodontics, Faculty of Dentistry, University of Seville, 41009 Seville, Spain; jmbmora@us.es; 3 Paediatric Dentistry, Faculty of Dentistry, University of Seville, 41009 Seville, Spain; amendoza@us.es (A.M.-M.); dribas@us.es (D.R.-P.)

**Keywords:** CAMBRA, dental caries, environment, parents, pH, prevention, risk of dental caries, sialometry

## Abstract

**Background/Objectives**: To explore the association between parental knowledge on dental caries prevention and the risk of caries in pediatric patients aged 6 to 14 years who reside in the province of Seville, using the CAMBRA preventive protocol as an assessment tool. **Methods**: After the approval granted by the Ethics Committee, a descriptive and analytical observational study was conducted. Caries risk was established using the CAMBRA Questionnaire, pH measurement, and salivary flow rate. To assess the socioeconomic background of the patients and their hygiene and dietary habits, parents completed two surveys: the first about the quality of the patient’s diet, and the second directly related to the CAMBRA questionnaire used and validated by the University of Seville. **Results**: The final study sample consisted of 300 pediatric patients, aged 6 to 14 years, of whom 54% were boys and 46% were girls. The caries risk distribution was as follows: 33% low, 7% moderate, 48.6% high, and 11.3%. A total of 61.7% of the participants live in urban areas, while 38.3% are from peri-urban regions. There is a statistically significant association between socioeconomic status and family circumstances in children with a risk of caries. Furthermore, an association was established between caries risk, dietary habits, and oral hygiene. **Conclusions**: Parental knowledge about dental caries prevention and caries risk in children was found to have a strong association with reduced caries risk in children.

## 1. Introduction

Historically, caries has been considered an infectious, chronic, transmissible, and dynamic disease caused by microorganisms. It is located in the hard tissues of the teeth and begins with demineralization of the enamel, caused by organic acids produced by certain oral bacteria when carbohydrates are metabolized in our diet. However, today, the concept of caries encompasses both the disease itself and its clinical manifestations. This condition involves dysbiosis in the normal oral biofilm, which reacts dynamically to sugar-rich diets, generating acids that cause caries lesions [1,2,3].

Dental caries is, therefore, a microbial disease that arises from an imbalance in the oral microbiome. This imbalance results in a shift in bacterial species, favoring those that produce or tolerate acids, increasing the risk and activity of caries. The current definition of caries establishes that it is a dynamic, chronic, non-communicable process mediated by bacteria and influenced by diet, which manifests clinically as the loss of minerals in the hard tissues of the tooth [4,5,6].

Preventive strategies focus on addressing each of the factors involved in the etiology of caries: the agent, the host, and the substrate. The dentist must select the most appropriate preventive measures for each situation, with the goal of preventing the development of caries and its potential consequences [1,4].

The primary objective of any caries intervention is to preserve healthy teeth and prevent the disease from developing; that is, to achieve primary prevention in the prepathogenic phase of the disease. However, this is not always possible due to the high prevalence of caries and the numerous sequelae associated with this pathology. Therefore, it is essential to develop guidelines for global risk assessment [7,8]. Risk factors play a crucial role in the etiology of caries; their presence directly increases the likelihood of disease development, while their absence or elimination decreases this probability [7,9].

The diagnosis of dental caries in children involves not only the identification and monitoring of carious lesions but also a variety of factors. This includes assessing caries risk, analyzing the natural history of caries progression, assessing and reassessing disease activity, evaluating the results of previous treatments, and parental expectations and preferences. Multiple tools have been developed to assess caries risk, including the CAMBRA protocol, the subject of this study [7,10].

The CAMBRA protocol, promoted by the California Dental Association (CDA) in 2002, is a procedure that analyzes a patient’s caries risk based on their age, assessing the type and amount of dental plaque present. The original model includes four risk levels: low, moderate, high, and extremely high. This questionnaire assesses the caries lesion and proposes action protocols based on risk level [7,10,11].

CAMBRA allows patients to be classified according to their risk assessment, considering caries prevalence, and establishes treatment protocols that offer an individualized approach for each risk group. This facilitates treatment focused on caries-related protective factors. Additionally, it plays a preventive and interceptive role in the disease, covering the entire population; even those considered low-risk can benefit from prevention recommendations [7,10,11,12].

However, the effectiveness of these individualized preventive strategies is closely related to behaviors established within the family environment. The caries risk outcome obtained through the CAMBRA protocol in pediatric patients largely depends on the daily habits promoted at home [13,14,15].

Parents play a crucial role in the behavior of a child in the pediatric dentist [13,14,15]. Recently, different parenting styles have gained great interest since parents, as primary caregivers, exert a significant influence on the development of their children’s present and future emotional health, personality, character, well-being, social and cognitive development, and academic performance. This is transferred to the dental office, affecting the interaction with the dentist [13]. Parenting styles also influence children’s health. Childhood obesity and sugar consumption are increasing among children, and this is a concern for children’s oral and general health [13,14]. Therefore, understanding the level of knowledge and the practices of parents and caregivers plays an important role in the advancement of intervention programs aimed at modifying behavior and promoting the improvement of oral health of child patients [14,15].

Prevention and treatment of childhood caries require a comprehensive approach that combines individualized clinical strategies, such as the CAMBRA protocol, with the essential role of the family in adopting healthy habits and shaping behaviors that promote good oral health from the early years [7,10,14,15].

## 2. Materials and Methods

### 2.1. Study Type and Settings

This cross-sectional, observational, and analytical study was approved by the Research Ethics Committee of the University of Seville. The sample consisted of 300 children who had no known allergies to any of the products used in the study. The sample size was calculated based on a risk of error (α) with a 95% confidence interval (CI) and a risk of error (β). Patients who underwent orthodontic treatment, systemic pathologies, or daily medication were excluded. The guardians consented to the use of their records and personal data for scientific purposes. Various types of data were collected.

### 2.2. CAMBRA Assessment Questionnaire

First, CAMBRA questionnaires were applied to obtain the caries risk of pediatric patients according to the CAMBRA protocol. Saliva samples were taken to measure pH, buffering capacity, and salivary flow rate. The four original levels of caries risk (low, moderate, high, and extreme) were considered, and based on these data, a dental education program on oral hygiene was implemented, and a diet analysis was performed to make the necessary recommendations about the foods that could promote the appearance of caries at a young age [7].

### 2.3. Clinical Examination

As part of the clinical assessment, the presence of bacterial plaque on dental surfaces was examined to determine the state of oral hygiene. The presence or absence of bacterial plaque on dental surfaces was evaluated using the modified Quigley–Hein plaque index as a reference. The measurement focused on the third gingival layer of the buccal and lingual dental surfaces of all erupted teeth [6]. The average index was obtained by dividing the sum of the total surface score by the number of surfaces examined. A score of 0 to 1 corresponded to low amounts of bacterial plaque, and 2 or more was associated with high plaque scores [16].

### 2.4. Saliva Analysis

To evaluate the salivary characteristics of the patients, specific tests were performed to measure salivary flow rate and pH. A sialometry test was performed by stimulation with paraffin chewing gum to measure salivary flow rate (SFV) in patients [17]. The result of this collection was expressed as milliliters per minute and was obtained by dividing the salivary volume by the number of minutes elapsed. A salivary flow rate of less than 1 mL/min in 5 min was considered hyposalivation [17,18]. Regarding salivary pH measurement, the pH scale was taken into account, which includes values between 0 and 14, through which the acidity or alkalinity of saliva was estimated. In the case of the salivary sample studied, which is composed of 99% water and 1% organic and inorganic molecules, the normal pH has a value between 6.7 and 7.4. That is, it is relatively neutral [18,19,20]. The value legend included in the GC Europe Saliva Check Buffer pack, which was the set purchased for sialometry and salivary pH measurement, was used as a reference.

### 2.5. Socioeconomic Data Collection

To assess parents’ knowledge and children’s socioeconomic background on dental caries prevention, parents completed two surveys. The first was to assess dietary habits using the Diet Quality Questionnaire of the Spanish Society of Dietetics and Food Sciences (SEDCA), which has been validated and used in previous studies with the Spanish pediatric population (Appendix A). This questionnaire has good internal consistency and sensitivity to identify dietary patterns that could influence oral health. This survey is validated for the Spanish child population and can produce three possible results: a good diet, a diet with room for improvement, and a poor diet [21].

The second survey was conducted to analyze knowledge and practices related to oral hygiene habits to prevent dental caries. This questionnaire was adapted from the CAMBRA protocol, but it underwent an additional validation process that included a pilot test with a representative sample of parents to assess the clarity of the questions. The questionnaires were administered by the same interviewer to minimize bias and ensure uniformity in data collection. Regarding socioeconomic background, data were collected on: age, gender, educational level, and nationality of parents, whether the child patients selected for the study were only children or not, whether their parents were separated, and whether the pediatric patients lived in an urban or peri-urban environment. In addition, 14 questions were included in their knowledge of dental caries prevention (Appendix B).

### 2.6. Radiographic Exam

All patient examination radiographs were obtained digitally following standard-of-care procedures. It is the standard of care to obtain caries detection radiographs if no diagnostic-quality radiographs have been available in the past year, according to the AAPD [22]. The principal investigator clinically and radiographically evaluated the patients, classifying the various pathology into different types: enamel caries, dentin caries, and pulpal caries.

The gold standard for inter-operator agreement was Dr. Asunción Mendoza-Mendoza, Professor of Pediatric Dentistry at the University of Seville, who has extensive experience in conducting studies of this nature.

### 2.7. Statistical Analysis and Validation

Regarding the database, a data collection sheet was used using Microsoft Excel, in which patient codes, full names, and values of the variables analyzed were recorded. To ensure the anonymity of the patients included in the study, each was assigned a numerical code. Only the principal investigator of this study had access to these data. A table was designed to record all data using SPSS v. 29 for analysis. To validate the collected data, a detailed verification process was conducted to ensure that all entries were correctly coded and that the information was complete and consistent. Incomplete or incorrect data were identified and removed from the database to avoid potential biases in the results. Subsequently, a comprehensive descriptive analysis was performed to characterize the sample based on the main sociodemographic and clinical variables. In addition, a more in-depth analysis was performed, including the application of multivariate models to identify significant associations between the variables studied and the risk of caries. This approach allowed for more robust results and allowed for adjustment for potential confounding factors.

## 3. Results

Within the sample, 162 patients were boys (54%) and 138 girls (46%). The age distribution shows a fairly uniform spread across the range from 6 to 14 years old, with slight variations between genders. In particular, younger age groups (6 to 8 years) had a higher proportion of boys compared to girls, while in some middle age groups (9 to 11 years), the proportion of girls was slightly higher or similar to boys. In older age groups (12 to 14 years), the numbers tend to level out again, with small differences between boys and girls. Overall, the data suggest that there was no significant gender skew in the sample across the age range studied, indicating a balanced representation for further analysis.

To determine whether the quantitative variables met the normality criteria, the Kolmogorov–Smirnov test with Lilliefors correction was performed; the assumption of normality was rejected based on the results obtained. Medians, percentiles, and interquartile ranges for all these variables were calculated, producing a median of 9 for age, with an interquartile range of 4. For stimulated salivary flow, the median was 8, with an interquartile range of 4. For salivary pH, the median was 7, with an interquartile range of 0.4. The frequencies and percentages of all the quantitative variables collected in the study were calculated (Table 1, Table 2, Table 3 and Table 4). The variables were divided into 4 tables, based on the grouping selected for each one.

The Table 1 shows the qualitative variables related to the risk of caries in the patients selected for the study. The table presents information on the following variables: caries risk determined by the CAMBRA questionnaire, the modified Quigley–Hein plaque index, diet quality, and sugar consumption more than three times a day. These results are divided in the table by gender of the selected sample (Table 1).

Sixty percent of children are at high or extreme risk for caries. Similar results were obtained for the variables used to determine the risk of caries, determined by CAMBRA. Almost 83% of children have some level of dental plaque. Although only 3.7% have a “poor” diet, 54.3% have a diet that could be improved. More than 70% of the children consumed excessive sugar (Table 1).

The following table presents information on the family situation or environment of the children included in the study. The qualitative variables selected were as follows: whether or not they were single children, the parents’ marital status (whether the parents were separated or not), and the children’s primary residence.,Seville, an urban area, or a periurban area (Table 2).

The vast majority, 79.3%, of the children had siblings. Therefore, 20.7% of the children were single children. Only 9.3% of the children had separated parents. 61.7% lived in the city of Seville, while 38.3% lived in peri-urban areas (Table 2).

**Figure 1:**
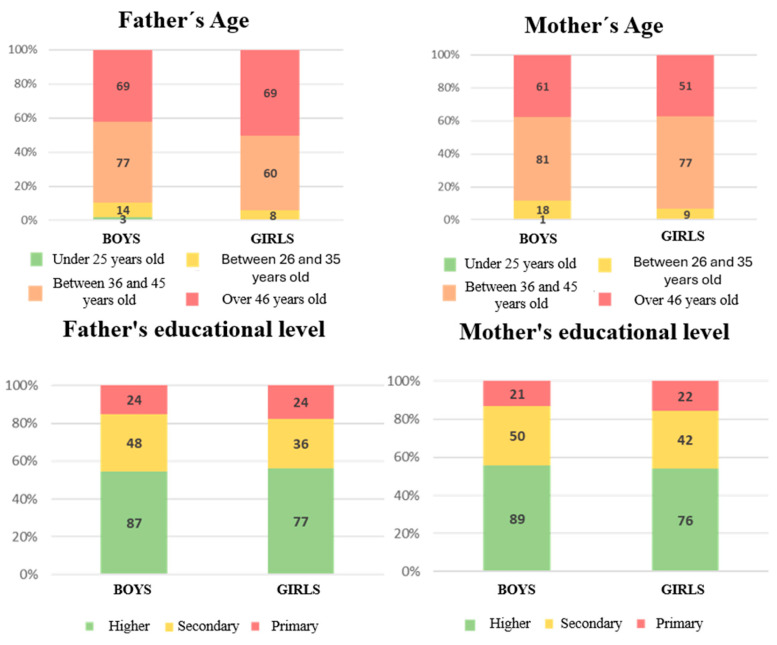
Frequencies and percentages of relevant qualitative variables referring to the socioeconomic environment of children.

Figure 1 shows information on the socioeconomic background of the 300 participants, determined by the age, educational level, and nationality of the parents. Most of the fathers were over 35 years old, with an almost equal distribution between the 35–45 age groups (45.7%) and those over 45 years old (46%). Regarding the mothers of the patients, most were between 35 and 45 years of age (52.7%), slightly younger than the fathers on average. Regarding the educational level, 54% of fathers and 55% of mothers had higher education.

However, a small percentage of mothers had primary education (14.3%). Regarding the nationality of the parents, most of the two sexes were Spanish (approximately 90%).

The following table shows the frequencies and percentages of responses that were included, as previously mentioned, in the survey of parents to determine their knowledge about dental caries prevention habits (Appendix B) (Table 3).

In general, high rates of affirmative responses were obtained, such as in Question 3 (86%) and Question 14 (80%). It should be noted that in Question 6, the majority of parents responded “No,” with 98.3%. In Question 11, the responses were split exactly 50/50 (Table 3).

In general, the proportion of responses does not vary significantly between boys and girls. However, we note that in Question 5, more parents of boys answered “Yes” (18.3%) compared to parents of girls (12.7%). However, in Question 12, the difference is also notable: More parents of boys (19.3%) answered “Yes” compared to parents of girls (14%) (Table 3).

Pearson’s chi-square test was performed to test the significance among categorical variables. Subsequently, we describe the significance of each categorical independent variable relative to the other categorical dependent variables. Caries risk (CAMBRA) is observed to be the indicator with the most statistically significant associations, especially with a history of cavities in caregivers, the presence of cavities in the child, the frequency of dental visits, frequent sugar consumption, and fluoride use, indicating that these factors are key determinants of caries risk. Daily sugar consumption also shows significant associations with most variables related to diet and fluoride use. In contrast, the plaque index was only significantly associated with the consumption of beverages other than water (Table 4).

The remaining results obtained from the Chi-square test for the independent variables did not show any significant association with the dependent variables. In the following, we detail the significance of the association between each categorical dependent variable and the other categorical dependent variables. The educational level of both parents, father, and mother, is the sociodemographic factor with the highest number of statistically significant associations with children’s oral health habits. It is primarily related to frequent sugar consumption, knowledge of fluoride, and the use of fluoride toothpaste. Some significant associations are also observed with the parents’ nationality and, to a lesser extent, with their age, especially in aspects such as supervising tooth brushing and the consumption of sugary drinks (Table 5 and Table 6).

The variables in which statistically significant results were obtained have been included in Table 4, Table 5 and Table 6. It is highlighted, for example, that there were no relevant results regarding the type of brush used by the patients.

Because the sample did not have a normal distribution, nonparametric statistical tests were applied, in this case, the Wilcoxon test for related samples. In the following, we detail the significance of the numerical independent variable (age) with the other numerically dependent variables (stimulated salivary flow and salivary pH). Both results showed a statistically significant relationship (*p* = 0.001). There was a significant association between the numerically dependent variables since the *p* value was less than 0.05 (*p* = 0.026).

Logistic regression analysis was used to identify variables significantly associated with the likelihood of the event of interest. This approach allows exploring how different factors contribute to the occurrence of the phenomenon studied, facilitating a better understanding of the collected data. The numerical variables were grouped into three groups: age, divided into three groups according to the type of dentition (young children with mixed dentition, phase 1: between 6 and 9 years; middle-aged children with mixed dentition, phase 2: between 10 and 12 years; older children with permanent dentition: between 13 and 14 years). Regarding the salivary pH variable, values were grouped into two intervals based on the type of pH (acidic pH, between 6.2 and 7; basic pH, between 7.2 and 7.8). Regarding the values of the stimulated salivary flow variable, they have been grouped into two ranges: values less than 5 mL/min are associated with hyposalivation, while values greater than 5 mL/min are associated with normal salivation.

In the following, with respect to age, using the oldest group (13–14 years) as a reference, significant associations were observed with the educational level of the father (secondary and high school) and with the responses to the preventive knowledge questions (questions 1 and 7) in the 6- to 9-year-old group. For children aged 10–12 years, significant associations were found with the educational level of the father (secondary and higher education), the age of the mother (26–35 years), and the responses to question 9 on brush frequency.

Salivary pH, taking the basic pH group as a reference, with the other categorical variables showed significance with the patient’s gender (*p* = 0.039 and OR = 0.560), the modified Quigley–Hein plaque index, variable 0 (*p* = 0.044 and OR = 0.082), the Patient’s Diet Quality Survey, good (*p* < 0.001 and OR = 2.065 × 10^−9^) and place of residence (*p* = 0.024 and OR = 2.019).

There is a statistically significant relationship in the stimulated salivary flow, taking as reference the normal salivary flow group, with the age of the male parent, between 26 and 35 years (*p* = 0.042 and OR = 0.049), question 3 (*p* = 0.007 and OR = 5.481), question 8 (*p* = 0.002 and OR = 0.051) and question 9 (*p* = 0.035 and OR = 0.112).

## 4. Discussion

From the analysis of the data obtained, relevant patterns were identified that may contribute to a better understanding of the multifactorial dynamics of this disease.

In this project, the CAMBRA questionnaire had to be adapted to clinical activities, so we eliminated the possibility of performing salivary bacterial cultures, as these were not feasible to perform and maintain in dental offices for financial and time reasons. The objective was to complete the questionnaires as realistically as possible for pediatric dentistry practice.

The sample consisted of 300 healthy patients between the ages of 6 and 14. The lower age limit was established for various methodological reasons. In particular, the CAMBRA (Caries Management by Risk Assessment) system, used as the reference tool in this research, distinguishes two age categories in its questionnaires: 0 to 5 years and 6 years and older. To maintain the homogeneity of the data collection instrument and ensure comparability between participants, the same questionnaire was used for all patients beginning at 6.

Regarding the studies found in the literature, there is a disparity in the age ranges and sample sizes of the study populations. Studies with a wide age range (from 3 to 17 years old) were reviewed. However, other publications focus their study sample on smaller age groups, such as those between 6 and 12 years of age.

In terms of methodology, the present study used the CAMBRA questionnaire and three additional validated surveys to collect information on oral hygiene and eating habits in pediatric patients, as well as data on their family and socioeconomic background.

### 4.1. Caries Risk Measurement

A high risk of caries was found, mainly measured by the CAMBRA questionnaire, with a prevalence of 48.7%. Iqbal et al. [23] and Aboubakr et al. [24] also measured caries risk, obtaining a moderate (61.2%) and a high (92.5%) caries risk, respectively. Other studies measured the prevalence of caries, obtaining a risk of 54.6% [25,26,27]. Although prevention programs have been implemented for years, data show that caries risk remains high in pediatric populations. Factors such as parental knowledge about oral health or oral hygiene appear relevant in this context. This suggests the need to explore more effective preventive and educational strategies aimed at both children and their primary caregivers.

Other articles measure the prevalence of caries in study populations using other methods. García-Pola et al. [26] do so based on the WHO diagnostic criteria (1997) [28]. Others, however, do so through the ICDAS criteria, as García et al. [29] did in 2021. In the study by Carmagnola et al. [30], 39% of patients up to 11 years of age had caries.

### 4.2. Oral Hygiene Status

Thirty-nine percent of the examined children had separate areas of bacterial plaque on the cervical margin of the tooth, which may be indicative of poor oral hygiene. This increases the likelihood of developing dental caries. Ellakany et al. [31] measured the gingival status of the children and found that 84.21% were free from gingivitis. The quality of the diet in 54.3% of the patients could be improved, with 71.7% consuming sugar more than three times a day.

### 4.3. Family Environment and Sociodemographic Level

Of the children, 79.3% had siblings, 90.7% of the parents of the patients were not separated, and 61.7% lived in the city of Seville. These data provide a general description of the family context of the sample. However, Fernández et al. [32] compared the prevalence of caries in different places of residence, comparing rural and urban areas, and found no statistically significant differences.

In relation to the age of the parents, the majority were between 36 and 45 years old, with 45.7% of fathers and 52.7% of mothers. The predominant educational level was higher education for both parents (54% and 55%, respectively). The majority of parents were of Spanish nationality, with 88.7% and 89.7%, respectively.

Chen et al. [33], Carmagnola et al. [30], Aboubakr et al. [24], Iqbal et al. [23], and Ellakany et al. [31] conducted a survey among parents of child patients with questions about eating habits and oral hygiene. In addition, they collected information about the socioeconomic environment of the patients [25,30,31,32,33].

They suggested that the socioeconomic level may influence the appearance of oral diseases and the related quality of life. They also found a statistically significant association between educational level and the age of parents, especially the mother of the children, and good health habits [31,32,33]. These data are in line with those observed in our work. Abbasoglu et al. [34] and Van Ligten et al. [35] did not find a significant relationship between the type of family model and the prevalence of caries. This is in contrast to our results, where we did not find a relationship. In the study by García-Pola et al. [26] and in that of Van Ligten et al. [35], dental caries were more common among immigrant children than among Spanish children.

### 4.4. Salivary Tests

Regarding the data obtained on the salivary environment of the oral cavity in pediatric patients, both stimulated salivary flow (sialometry) and salivary pH showed statistically significant associations with each other and with several categorical variables. The median values for stimulated salivary flow were 8 mL/min and 7 mL/min, respectively. These values are substantially higher than the clinically accepted cut-off point for hyposalivation (≤0.7 mL/min for stimulated flow) [17,18], indicating that, in general, the studied population does not present hyposalivation. Similarly, the median salivary pH was within neutral values (pH ≈ 7), which is generally recognized as a protective factor against enamel demineralization and caries development [17,20,36].

However, it is important to note that patients with acidic salivary pH values (≤6.5) demonstrated statistically significant associations with higher Quigley–Hein plaque index scores, lower dietary quality, and specific places of residence. This finding suggests that environmental and behavioral factors may be exerting a negative influence on the acid-base balance of the oral environment, increasing the risk of caries even when salivary flow remains within normal parameters.

In relation to hyposalivation, although the median values indicated adequate salivary secretion, statistically significant associations were identified with parental characteristics (specifically, the age of the male parent between 26 and 35 years), as well as with oral hygiene behaviors such as having pediatric dental check-ups, daily use of 1450 ppm fluoride toothpaste, and brushing three times per day. These findings suggest that salivary function may be indirectly influenced by family dynamics, health behaviors, and socio-environmental factors that require further investigation.

When contextualizing these results with the available literature, it is notable that only a limited number of recent studies have specifically addressed the relationship between salivary parameters and caries risk in pediatric populations. The work of Choudhary et al. [37] and Iqbal et al. [23] are among the few that support a strong association between salivary pH, buffering capacity, and caries prevalence. However, the generally neutral salivary pH and absence of hyposalivation in this study contrast with the populations studied by those authors, where reduced salivary buffering and pH were more prevalent, likely due to differing sociodemographic and environmental contexts. These authors also did not take into account the count of S. mutans and lactobacilli bacteria in saliva.

Therefore, while our findings are partially consistent with previous research on the role of salivary pH in caries risk, they also highlight the particular importance of behavioral and environmental factors over purely physiological salivary parameters in this specific population. Future research should adopt standardized diagnostic criteria for salivary evaluation and include stronger comparisons between populations to clarify the relative contributions of biological and behavioral risk factors in pediatric oral health.

### 4.5. Parents’ Prevention Knowledge

Based on the results obtained through the collection of data using the prevention knowledge survey, we will discuss some of the most relevant and striking findings. In particular, we will address aspects in which results showed similarities with previous studies, allowing comparisons and reinforcing the validity of our observations. Likewise, we will highlight specific issues for which no references were found in the consulted literature, underscoring the originality and added value of this study.

In total, 86% of the children had a dental visit in the last year, a figure significantly higher than in previous studies (54.8%), and this percentage was higher among children whose parents had higher educational levels and were older, diet quality, and lower risk of caries. These results exceed those reported by García et al. [29], where 54.8% of children visited the dentist every six months.

However, 69% of children do not regularly consume beverages other than water; however, those who do show a higher risk of caries are associated with socioeconomic factors and diet habits. This indicates that frequent dental check-ups alone may not suffice to reduce the risk of caries if they are not accompanied by adequate dietary habits. In the study by García et al. [29], 55.6% consumed beverages other than water at least once a day. Kumar et al. [25] found that 70% of children with caries consumed sugary drinks more than twice a day. Furthermore, nearly 88% of children with caries consumed solid sugar more than twice a day Similar results were found in other studies [38,39,40].

Regarding oral hygiene, 4% of parents are unaware of the role of fluoride, representing an alarming educational gap that directly impacts caries prevention. Parental knowledge about fluoride use was low despite the high frequency of brushing, highlighting the discontinuity between attending dental check-ups and understanding the most effective preventive tools. None of the reviewed articles considered this information, yet it is relevant because it emphasizes the importance of implementing oral health educational campaigns aimed at the population, especially parents. Fluoride has been shown to have a positive influence on the prevention of dental caries [39,40,41].

In addition, 82% of children use fluoride toothpaste daily at 1450 ppm, but misinformation and misuse persist, limiting its preventive effectiveness. Carmagnola et al. [30] reported that caregivers stated their children brushed at least once a day in approximately 80% of cases. However, in the study by Kumar et al. [25], 43% of parents did not know what type of toothpaste their children used. Additionally, 5% did not use fluoride products at all, despite the fact that controlled exposure to fluoride has been proven to be a clear protective factor against caries [27]. In the study by Ellakany et al. [31], 44.48% of the patients did not use fluoride toothpaste. It is recommended that educational strategies on oral hygiene focus not only on brushing frequency and product awareness but also on the correct use of these preventive tools.

Furthermore, 74.7% of children do not brush three times a day before the age of 6 years and, although no association was found with other factors, this reflects an insufficient habit during a critical age for prevention. Carmagnola et al. [30] found that 24% of children started brushing after tooth eruption, while 7% started after age 4. García et al. [29] found a statistically significant association between brushing frequency (two times a day) and caries prevalence.

This shortcoming becomes more serious considering that 50% of parents do not supervise their children’s daily brushing, a factor that has been associated with a higher risk of caries, highlighting the need for active caregiver involvement. In the study by Carmagnola et al. [30] in 2020, 20% and 53% of children received help brushing always or sometimes, respectively.

Finally, 72.3% of children do not use fluoride mouthwash daily, a worrying figure directly related to maternal educational level and parental nationality. Recent studies, such as that by Iqbal et al. [23], show that frequent use of fluoride mouthwash is associated with a lower incidence of caries, highlighting a significant opportunity to improve prevention through family education.

If we compare all these data with the high risk of caries and the high prevalence of existing cavities, it follows that tooth brushing must be optimal and frequent. It is important to properly supervise children’s tooth brushing, especially at early ages.

It is important to note that there are significant gaps in knowledge about prevention among parents of child patients, which in practice translates into a high risk of dental caries in children. Social outreach and training programs on proper oral health habits should be established for the children’s community.

This discussion seeks not only to interpret the data but also to propose potential new lines of research and possible intervention strategies to prevent dental caries in populations with similar characteristics. We believe that it is important to implement a greater number of health promotion interventions, both from a dietary perspective and in terms of oral hygiene education for the entire family.

Parents, as primary caregivers for their children’s daily care, directly influence the establishment of oral hygiene habits, the adoption of a healthy diet, and access to dental care. A higher level of parental knowledge is associated with better preventive practices, such as proper brushing, sugar control, and regular pediatric dental check-ups. It has also been observed that parents’ educational and socioeconomic status can influence their children’s oral health, highlighting the need to develop education for families, especially in vulnerable settings. In this sense, oral health education, especially for parents, appears to be a relevant strategy to reduce the prevalence of caries in pediatric patients and improve their daily quality of life.

## 5. Conclusions

We can conclude that there is strong evidence between parental knowledge on the prevention of dental caries and the risk of caries in child patients. Higher parental awareness of oral health may act as a protective factor against the development of caries in childhood. Therefore, strengthening preventive education programs in this regard is essential to promote this significant association.

## 6. Limitations

This study has a limitation that should be considered when interpreting the results: the lack of randomization in the patient sample selection. There is also a lack of longitudinal follow-up, as it is not possible to determine the long-term impact of interventions or parental knowledge on children’s oral health.

## 7. Future Directions

We believe that this work opens up many different lines of research. New studies should be conducted in the future, with larger and more diverse samples, to evaluate the long-term impact of educational interventions on children’s oral health. Future research lines should especially consider children with special needs, illnesses, or the impact of medications, including several operators who validate the objectivity of this new pilot protocol. Children with behavioral pathologies, for example, developmental disorders or learning difficulties, require interventions with particularly reinforced preventive measures on an individual basis. Future studies are needed to evaluate the long-term impact of educational interventions on children’s oral health.

## Figures and Tables

**Table 1:** Frequencies and percentages of qualitative variables related to the risk of caries in children.

Variable		Boys	Girls	Total
		Frequency (%)	Frequency (%)	Frequency (%)
Gender		162 (54)	138 (46)	300 (100)
Risk of caries CAMBRA	Low	52 (32.1)	47 (34.1)	99 (33)
	Moderate	11 (6.8)	10 (7.2)	21 (7)
	High	81 (50)	65 (12.3)	146 (48.7)
	Extreme	17 (10.5)	17 (5.7)	34 (11.3)
Modified Quigley–Hein plaque index	0	22 (13.6)	30 (21.7)	52 (119)
	1	60 (37)	59 (42.8)	119 (39.7)
	2	62 (38.3)	40 (29)	102 (34)
	3	18 (11.1)	9 (6.5)	27 (9)
	4	0 (0)	0 (0)	0 (0)
	5	0 (0)	0 (0)	0 (0)
Diet Quality Survey	Good	61 (37.7)	56 (40.6)	117 (39)
	Improvable	93 (57.7)	79 (57.2)	172 (54.3)
	Bad	8 (4.9)	3 (2.2)	11 (3.7)
Sugar consumption more than 3 times/day	Yes	116 (71.6)	99 (71.7)	215 (71.7)
	No	46 (28.4)	39 (28.3)	85 (28.3)

**Table 2:** Frequencies and percentages of variables on the family situation or the environment of children.

Variable	Boys	Girls	Total	
	Frequency (%) Yes/No	Frequency (%) Yes/No	Frequency (%) Yes/No	Frequency (%) Yes/No
Single child	33 (20.4)	29 (21)	62 (20.7)	300 (100)
	129 (79.6)	109 (79)	238 (79.3)	
Separated parents	18 (11)	10 (7.2)	28 (9.3)	300 (100)
	144 (88.9)	128 (92.8)	272 (90.7)	
Place of residence	92 (56.8)	93 (67.4)	185 (61.7)	300 (100)
	70 (43.2)	45 (32.6)	115 (38.3)	

**Table 3:** Frequencies and percentages of variables in the survey to determine the knowledge of children’s parents about dental caries prevention.

Variable	Boys	Girls	Total	
	Frequency (%) Yes/No	Frequency (%) Yes/No	Frequency (%) Yes/No	Frequency (%) Yes/No
1. Has the mother or primary caregiver had cavities in the past year?	51 (17)/111 (37)	56 (18.7)/82 (27.3)	107 (35.7)/193 (64.7)	300 (100)
2. Has your child had fillings in the past year?	61 (20.3)/101 (33.7)	62 (20.7)/76 (25.3)	123 (41)/177 (59)	300 (100)
3. Has your child had dental visits in the last year?	138 (46)/24 (8)	120 (40)/18 (6)	258 (86) 42 (14)	300 (100)
4. Does your child eat snacks or drink sugary drinks between meals more than three times a day?	25 (8.3)/136 (45.3)	25 (8.3)/113 (37.7)	50 (16.6)/249 (83)	299 (100)
5. Does your child regularly drink other beverages than water?	55 (18.3)/107 (35.7)	38 (12.7)/100 (33.3)	93 (31)/207 (69)	300 (100)
6. Does your child sleep in with a bottle or breastfeed on demand while sleeping between the ages of 2 and 6?	3 (1)/159 (53)	2 (0.7)/136 (45.3)	5 (1.7)/295 (98.3)	300 (100)
7. Do you know the purpose of fluoride?	118 (39.3)/44 (14.7)	104 (34.7)/34 (11.3)	222 (26)/78 (74)	300 (100)
8. Does your child brush their teeth with 1450 ppm fluoride toothpaste daily?	131 (43.7)/31 (10.3)	115 (38.3)/23 (7.7)	246 (82)/54 (18)	300 (100)
9. Does your child brush their teeth three times a day?	44 (14.7)/118 (39.3)	44 (14.7)/94 (31.3)	88 (29.3)/212 (70.7)	300 (100)
10. Did your child brush their teeth three times a day before the age of 6?	38 (12.7)/124 (41.3)	38 (12.7)/100 (33.3)	76 (25.3)/224 (74.7)	300 (100)
11. Do you check your child’s brushing at least once a day?	87 (29)/75 (25)	63 (21)/75 (25)	150 (50)/150 (50)	300 (100)
12. Did you check your child’s brushing at least once a day before age 6?	58 (19.3)/104 (34.7)	42 (14)/96 (32)	100 (33.3)/200 (66.7)	300 (100)
13. Does your child use fluoride mouthwash or rinse?	44 (14.7)/118 (39.3)	39 (13)/99 (33)	83 (27.7)/217 (72.3)	300 (100)
14. Does your child use a manual toothbrush?	128 (42.7)/34 (11.3)	112 (37.3)/26 (8.7)	240 (80)/60 (20)	300 (100)

**Table 4:** Chi-square analysis evaluating the association between parental knowledge of prevention (based on survey data) and qualitative variables related to children’s caries risk.

Question	Modified Quigley–Hein Plaque Index	Caries Risk CAMBRA	Diet Quality Survey	Sugar Consumption Greater Than 3 Times/Day
1. Has the mother or primary caregiver had cavities in the past year?	0.155	0.003 *	0.001 *	0.001 *
2. Has your child had fillings in the past year?	0.001 *	0.001 *	0.016 *	0.005 *
3. Has your child had dental visits in the last year?	0.148	0.027 *	0.001 *	0.001 *
4. Does your child eat snacks or have sugary drinks between meals more than three times a day?	0.214	0.002 *	0.001 *	0.013 *
5. Does your child regularly drink other beverages than water?	0.001 *	0.003 *	0.001 *	0.001 *
7. Do you know the purpose of fluoride?	0.538	0.001 *	0.132	0.038 *
8. Does your child brush their teeth with 1450 ppm fluoride toothpaste daily?	0.162	0.015 *	0.003 *	0.015 *
11. Do you check your child’s brushing at least once a day?	0.299	0.009 *	0.305	0.898

* *p* value < 0.05 means statistical significance.

**Table 5:** Chi-square test relating the survey on parental knowledge of prevention to qualitative variables concerning the children’s sociodemographic environment.

Question	Education Level Male Parent	Education Level Female Parent	Nationality Male Parent	Nationality Female Parent	Age Male Parent	Age Female Parent
1. Has the mother or primary caregiver had cavities in the past year?	0.002 *	0.001 *	0.003 *	0.001 *	0.036 *	0.105
2. Has your child had fillings in the past year?	0.052	0.019 *	0.695	0.911	0.114	0.223
3. Has your child had dental visits in the last year?	0.039 *	0.055	0.356	0.464	0.603	0.014 *
4. Does your child eat snacks or have sugary drinks between meals more than three times a day?	0.001 *	0.001 *	0.738	0.356	0.022 *	0.001 *
5. Does your child regularly drink other beverages than water?	0.001 *	0.001 *	0.011 *	0.569	0.103	0.001 *
7. Do you know the purpose of fluoride?	0.001 *	0.001 *	0.084	0.010 *	0.446	0.722
8. Does your child brush their teeth with 1450 ppm fluoride toothpaste daily?	0.001 *	0.001 *	0.373	0.091	0.001 *	0.001 *
11. Do you check your child’s brushing at least once a day?	0.056	0.087	0.069	0.343	0.002 *	0.004 *
13. Does your child use fluoride mouthwash or rinse?	0.221	0.012 *	0.023 *	0.021 *	0.474	0.669

* *p* value < 0.05 means statistical significance.

**Table 6:** Chi-square test relating the survey on parental knowledge of prevention to qualitative variables concerning the children’s sociodemographic environment.

Question	Single Child	Place of Residence	Parents Separated
1. Has the mother or primary caregiver had cavities in the past year?	0.309	0.048 *	0.404
2. Has your child had fillings in the past year?	0.005 *	0.242	0.155
3. Has your child had dental visits in the last year?	0.490	0.182	0.272
4. Does your child eat snacks or have sugary drinks between meals more than three times a day?	0.004 *	0.013 *	0.078
5. Does your child regularly drink other beverages than water?	0.391	0.001 *	0.064
7. Do you know the purpose of fluoride?	0.969	0.167	0.218
8. Does your child brush their teeth with 1450 ppm fluoride toothpaste daily?	0.292	0.001 *	0.311
11. Do you check your child’s brushing at least once a day?	0.254	0.044 *	0.112
13. Does your child use fluoride mouthwash or rinse?	0.364	0.455	0.059

* *p* value < 0.05 means statistical significance.

## Data Availability

Original contributions presented in this study are included in the article and Supplementary Materials. Further inquiries can be directed to the corresponding author.

## Supplementary Materials

The following supporting information can be downloaded at https://www.mdpi.com/article/10.3390/children12070824/s1, Table S1: Chi-square analysis assessing the association between qualitative variables referring to the socioeconomic environment of children and variables related to children’s caries risk; Table S2: Chi-square analysis assessing the association between between qualitative variables referring to the socioeconomic environment of children and variables related to children’s caries risk; Table S3: Kolmogorov–Smirnov test with Lilliefors correction for quantitative variables; Table S4: Median and interquartile range of the quantitative variables; Table S5: Percentiles of the quantitative variables with Tukey’s test. Figure S1: Random distribution of the sample’s age; Figure S2: Random distribution of stimulated salivary flow in the sample; Figure S3: Random distribution of salivary pH in the sample; Figure S4: Graph showing the frequency of females in the survey on parental knowledge about prevention; Figure S5: Graph showing the frequency of males in the survey on parental knowledge about prevention.

## Appendix A. Validated Survey on Diet Quality by the Spanish Society of Dietetics and Food Sciences

**DIET QUALITY SURVEY (SEDCA)**		
**QUESTION**	**YES**	**NO**
1. Do you consume at least 2 to 3 servings of fresh fruit every day?		
2. Do you regularly include a full serving of vegetables and greens in both lunch and dinner (making up about 1/3 to 1/2 of the plate)?		
3. Do you consume at least 2–3 servings of legumes per week?		
4. Do you regularly include raw or roasted (not fried or salted) nuts or raw or roasted seeds (such as chia, flaxseed, sesame, sunflower, pumpkin…) in your diet several times a week?		
5. When you eat breakfast cereals, bread, pasta, or flour products, do you choose whole grain versions instead of white or refined ones?		
6. Do you consume at least 3–4 servings of fish per week (including canned fish), with some of them being oily fish?		
7. Do you regularly eat sugary breakfast cereals, cookies, pastries, or sweets (more than 3 times a week on average)?		
8. Do you drink soft drinks, whether sugary or in their light/zero versions, several times a week (more than 2 soft drinks per week)?		
9. When buying packaged foods, do you read the nutrition label to choose the best options (no added sugars, quality fats, lower in salt, whole grain flours…)?		
10. Do you consume more than 4–5 servings of meat and processed meat products (cold cuts, sausages, meat preparations…) per week?		
11. Do your breakfasts usually include sugary cereals, cookies, commercial jams, sweetened cocoa powder, sugar, pastries…?		
12. Do you usually add sugar to foods such as coffee or yogurt, or choose their pre-sweetened versions?		
13. Do you often use refined oils (such as sunflower, palm, palm kernel…) for cooking or frequently buy products that contain those (precooked meals, cookies, breakfast cereals…)?		
14. Do you often skip meals to reduce your calorie intake, eat as little as possible, or follow very restrictive diets to the point of feeling very hungry at the next meal, experiencing food anxiety, or feeling unwell?		
15. Do you skip meals or snack on anything between meals to avoid taking a break at work or due to a lack of time to prepare proper meals?		
16. Do you often resort to quick dinners made with low-nutritional-quality processed foods, order fast food, or eat cold cuts, cookies with milk, or similar (2 or more times per week)?		

If your score is:

- Between 11 and 16 points: The quality of the diet is quite good. It is important to support an active lifestyle and exercise.
- Between 6 and 10 points: The quality of the diet could be improved. Some of the points should be reviewed for improvement, and alternatives should be sought to improve them.
- Between 0 and 5 points: The quality of the diet needs to be improved. It is important to review the recommendations that are not being followed and to try to gradually introduce improvements to the diet.

## Appendix B. Validated Survey on Daily Dietary Habits of Pediatric Patients

**PARENTAL SURVEY ON ORAL HEALTH PREVENTION**	
This survey is a response to research being conducted at the University of Seville to determine the knowledge of parents of pediatric patients regarding hygiene and caries prevention measures.	
Age of 1st parent: ◦ Under 25 years old ◦ Between 25 and 35 years old ◦ Between 35 and 45 years old ◦ Over 45 years old	Age of 2nd parent: ◦ Under 25 years old ◦ Between 25 and 35 years old ◦ Between 35 and 45 years old ◦ Over 45 years old
Gender of 1st parent: ◦ Female ◦ Male ◦ Other	Gender of 2nd parent: ◦ Female ◦ Male ◦ Other
Educational level of 1st parent: ◦ Primary education ◦ Secondary education ◦ Higher education ◦ Don’t know, no answer (DK/NA)	Educational level of 2nd parent: ◦ Primary education ◦ Secondary education ◦ Higher education ◦ Don’t know, no answer (DK/NA)
Nationality of 1st parent:	Nationality of 2nd parent:

Is the patient an single child?	NO YES
Are the patient’s parents separated/divorced?	NO YES
Does the patient live in an urban setting?	NO YES
1. Has the mother or primary caregiver had cavities in the past year?	NO YES
2. Has your child had fillings in the past year?	NO YES
3. Has your child visited the dentist in the past year?	NO YES
4. Does your child eat snacks or sugary drinks between meals more than three times a day?	NO YES
5. Does your child regularly drink other beverages than water?	NO YES
6. Does your child sleep with a bottle or breastfeed on demand while sleeping between the ages of 2 and 6?	NO YES
7. Do you know the purpose of fluoride?	NO YES
8. Does your child brush their teeth with 1450 ppm fluoride toothpaste daily?	NO YES
9. Does your child brush their teeth three times a day?	NO YES
10. Did your child brush their teeth three times a day before the age of 6?	NO YES
11. Do you review your child’s brushing habits at least once a day?	NO YES
12. Did you review your child’s brushing habits at least once a day before age 6?	NO YES
13. Does your child use fluoride-free mouthwash or rinse?	NO YES
14. Does your child use a manual toothbrush?	NO YES
