# Supplementary material for: Impact of Parental Knowledge on Prevention Risk of Caries in Seville Children Between 6 and 14 Years Old, Applying the CAMBRA Protocol
Source: Children (Basel). 2025 Jun 23;12(7):824. doi: 10.3390/children12070824 (PMC12293717; doi:10.3390/children12070824)
Supplement: Supplementary file 1 [file children-12-00824-s001.zip › children-3697777-supplementary.pdf]

QUESTION	MODIFIED QUIGLEY-HEIN PLAQUE INDEX		CARIES RISK CAMBRA		DIET QUALITY SURVEY		SUGAR CONSUMPTION GREATER THAN 3 TIMES/DAY	
	p	Cramér´s V	p	Cramér´s V	p	Cramér´s V	p	Cramér´s V
EDUCATION LEVEL MALE PARENT	0,004*	0,180	0,001*	0,191	0,001*	0,229	0,022*	0,160
EDUCATION LEVEL FEMALE PARENT	0,002*	0,185	0,008*	0,171	0,001*	0,331	0,001*	0,260
AGE MALE PARENT	0,390		0,134		0,152		0,482	
AGE FEMALE PARENT	0,212		0,092		0,001*	0,231	0,018*	0,184
NATIONALITY MALE PARENT	0,261		0,476		0,026*	0,156	0,287	
NATIONALITY FEMALE PARENT	0,607		0,217		0,014*	0,169	0,241	
	*p<0,05		*p<0,05		*p<0,05		*p<0,05	

TABLE S1. Chi-square analysis assessing the association between qualitative variables referring to the socioeconomic environment of children and variables related to children's caries risk.

QUESTION	MODIFIED QUIGLEY-HEIN PLAQUE INDEX		CARIES RISK CAMBRA		DIET QUALITY SURVEY		SUGAR CONSUMPTION GREATER THAN 3 TIMES/DAY	
	p	Cramér´s V	p	Cramér´s V	p	Cramér´s V	p	Cramér´s V
SINGLE CHILD	0,006*	0,203	0,008*	0,200	0,596		0,560	
PARENTS SEPARATED	0,016*	0,185	0,147		0,127		0,196	
PLACE OF RESIDENCE	0,116		0,035*	0,169	0,003*	0,198	0,141	
	*p<0,05		*p<0,05		*p<0,05		*p<0,05	

TABLE S2. Chi-square analysis assessing the association between between qualitative variables referring to the socioeconomic environment of children and variables related to children's caries risk.

VARIABLE	KOLMOGOROV-SMIRNOV TEST	
	**Sig.**	**Interpretation**
Age	< 0,001	Non-normal distribution
Stimulated salivary flow	< 0,001	Non-normal distribution
Salival pH	< 0,001	Non-normal distribution

TABLE S3. Kolmogorov–Smirnov test with Lilliefors correction for quantitative variables.

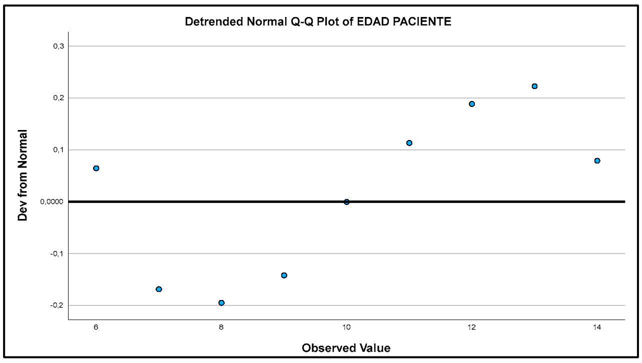

Figure S1. Random distribution of the sample's age.

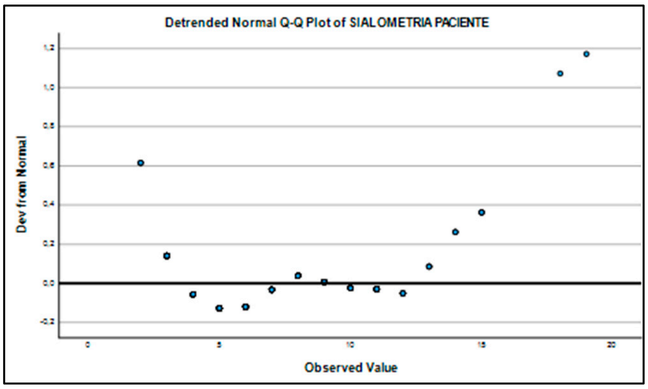

FIGURE S2. Random distribution of stimulated salivary flow in the sample.

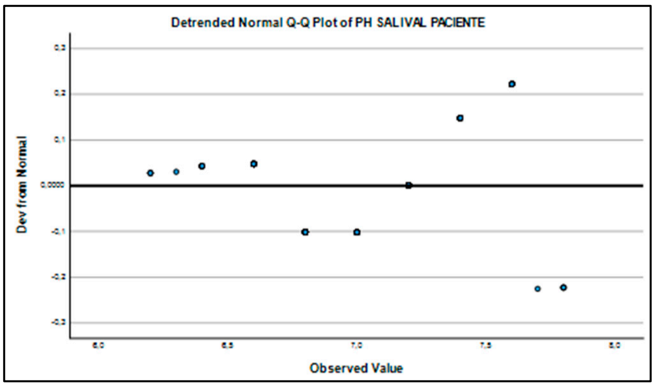

FIGURE S3. Random distribution of salivary pH in the sample.

VARIABLE	TOTAL	
	**Median**	**Intercuartile range**
Age	9	4
Stimulated salivary flow	8	4
Salival pH	7	0,4

TABLE S4. Median and interquartile range of the quantitative variables.

TUKEY´S TEST	VARIABLE	PERCENTILES						
		5	10	25	50	75	90	95
	Age			7	9	11		
	Stimulated salivary flow			5	8	9		
	Salival pH			6,8	7	7,2		

TABLE S5. Percentiles of the quantitative variables with Tukey's test.

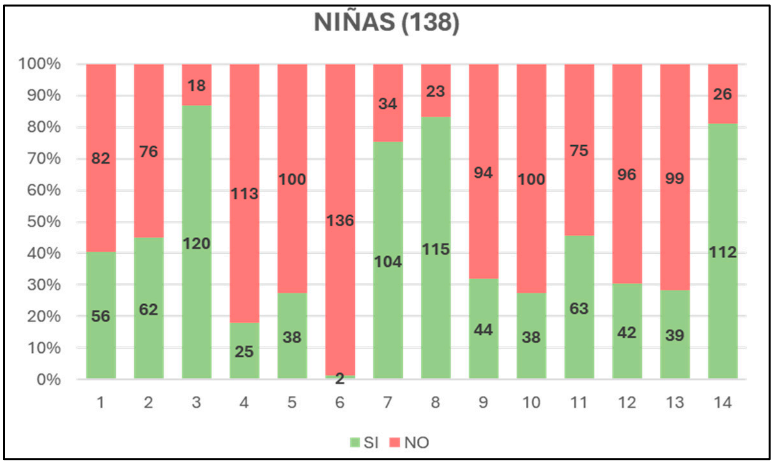

FIGURE S4. Graph showing the frequency of females in the survey on parental knowledge about prevention.

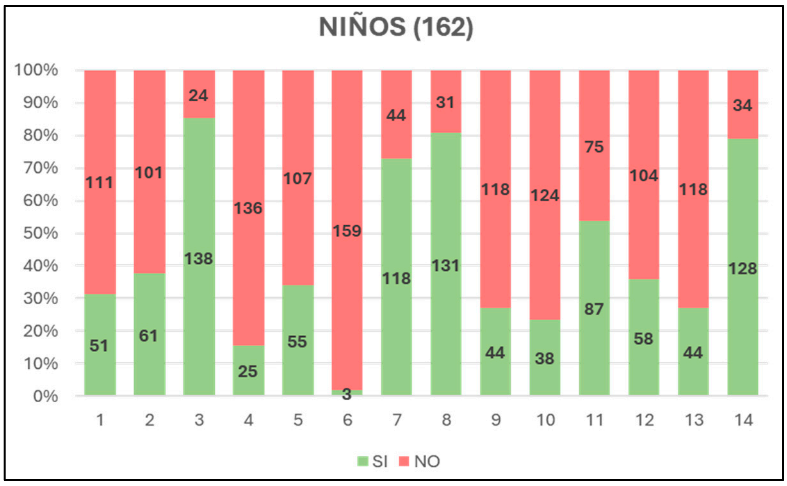

FIGURE S5. Graph showing the frequency of males in the survey on parental knowledge about prevention.
